# Comparative Evaluation of Novel Intracanal Medicaments on the Sealing Integrity of the Root Canal System: An In Vitro Study

**DOI:** 10.7759/cureus.72424

**Published:** 2024-10-26

**Authors:** Prerna Barge, Ravindra J Jadhav, Sabina Shaikh, Ajay S Kadam, Sandeep Y Sidral

**Affiliations:** 1 Pediatric and Preventive Dentistry, Yogita Dental College and Hospital, Khed, IND; 2 Conservative Dentistry and Endodontics, Vasantdada Patil Dental College and Hospital, Sangli, IND; 3 Conservative Dentistry and Endodontics, D Y Patil Dental School, Pune, IND; 4 Conservative Dentistry and Endodontics, Dr. Kadam's Multispeciality Dental Clinic, Pune, IND

**Keywords:** intracanal medicaments, metapaste, nano-particles, ncao, nmgo, nzno, qualitative and qualitative assay, sealing integrity, spectrophotometer, stereomicroscope

## Abstract

Aim and objective: This study aims to evaluate and compare the sealing integrity of endodontically treated teeth after the placement of novel nanoparticle-based intracanal medicaments through dye penetration.

Materials and methods: Total samples (n=56) with different experimental intracanal medicaments group 1: nano calcium oxide (nCaO) + propylene glycol (PPG) 400, group 2: nano zinc oxide (nZnO) + PPG400, group 3: nano magnesium oxide (nMgO) + PPG400, group 4: metapaste (control) were randomly divided for qualitative (n=28) and quantitative (n=28) analysis to determine the sealing integrity. The statistical analysis was performed using ANOVA, student t-test, and IBM SPSS Statistics for Windows, Version 20 (Released 2011; IBM Corp., Armonk, New York, USA), followed by determining sealing integrity using qualitative assay and quantitative assay.

Results: Highly significant differences (p<0.0001) were observed between all four groups during intragroup comparison for qualitative assay. The nCaO-based intracanal medicament showed the maximum linear measurements of 2% methylene blue dye penetrated compared to metapaste. However, intragroup comparison for quantitative assay had non-significant differences (p<0.0001).

Conclusion: All intracanal medicaments allowed some leakage to occur. Minimum dye penetration was observed with the metapaste group (control) followed by nano zinc oxide (nZnO) + PPG400, nano magnesium oxide (nMgO) + PPG400, and maximum dye was absorbed with nano calcium oxide (nCaO) + PPG400.

## Introduction

The long‐term success of endodontic therapy is directly related to the complete elimination of endodontic pathogens. The foremost microorganisms responsible for causing pulpal infections are anaerobic bacteria, of which the most resistant is *Enterococcus faecalis* (*E. faecalis*), followed by *Streptococcus mitis*, *Streptococcus sanguinis*, *Actinomyces* species, *Fusobacterium*, *Spirochetes*, and *Prevotella *species. Studies have proved that endodontic bacteria can penetrate up to a depth of 300-1500 µm within dentinal tubules [1].

It becomes impossible for routine chemo-mechanical aids to completely eradicate bacteria, their byproducts, and pulpal remnants from infected root canal systems which further necessitates the need for intracanal medicaments. Traditionally, calcium hydroxide-based intracanal medicaments were considered an integral part of the success of root canal therapies. The incomplete removal of intracanal medicaments from the root canals might compromise the sealing quality after obturation [2]. In the modern era, nanoparticles (NPs), owing to their smaller molecule size (1-100 nm), enhance surface area‑to‑mass ratios, heighten chemical reactivity, and provide a superior antibacterial effect when used in endodontics. Therefore, the present study aims to evaluate and compare the sealing integrity of endodontically treated teeth after the placement of novel NP-based intracanal medicaments. The null hypothesis was postulated that these novel NPs based intracanal medicaments had no difference in sealing the integrity of the root canal system.

## Materials and methods

This in vitro study was carried out in the Department of Pediatric and Preventive Dentistry, School of Dental Sciences, Krishna Vishwa Vidyapeeth, Karad, India, after obtaining the ethical clearance from the Institutional Ethics Committee, Krishna Institute of Medical Science (Deemed to Be University) (protocol number 163/2019-2020). The sample size (n=56) was obtained using G*Power software (latest ver. 3.1.9.7; Heinrich-Heine-Universität Düsseldorf, Düsseldorf, Germany), where a 95% confidence level and 95% power were considered to get a statistically significant result.

Inclusion criteria included single-rooted human teeth that are morphologically intact with non-carious roots, non-hypoplastic, or non-restored which were extracted for therapeutic purposes. The teeth that show morphological variation, fractures, hypoplastic lesions, and external resorption were excluded. The study materials (intracanal medicaments) considered while evaluating the sealing integrity of root canal-treated teeth were as follows: group 1: nano calcium oxide (nCaO) + propylene glycol (PPG) 400; group 2: nano zinc oxide (nZnO) + PPG400; group 3: nano magnesium oxide (nMgO) + PPG400; and group 4: metapaste (control). All the study materials and the vehicle (PPG400) were procured from Sigma-Aldrich.

Sample preparation

**Figure 1:**
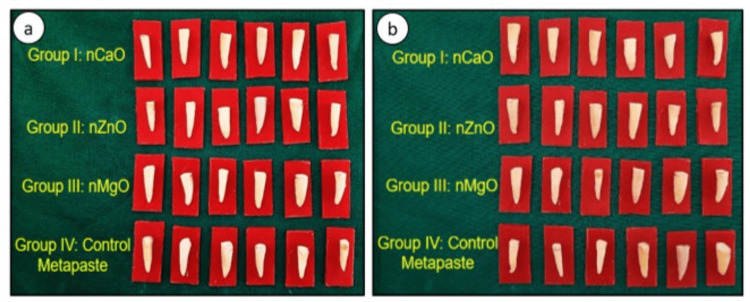
Pictorial representation showing randomization of teeth samples for (a) qualitative assay and (b) quantitative assay to determine sealing integrity using test materials: group 1: nCaO+PPG400; group 2: nZnO+PPG400; group 3: nMgO+PPG400; group 4: metapaste (control) nCaO: nano calcium oxide; nZnO: nano zinc oxide; nMgO: nano magnesium oxide; PPG400: polypropylene glycol 400 (vehicle)

Freshly extracted single-rooted human teeth samples were washed and cleaned under running tap water, followed by ultrasonic scaling to remove any remaining tissue remnants on the tooth surface. The teeth were examined under magnifying loops to rule out fractures or defects. For standardization of samples, the radicular part of the tooth was sectioned in a transverse plane 15 mm from the apex toward the coronal part of the tooth using NTI® diamond discs. These 56 sectioned teeth samples were stored in normal saline until use. For biomechanical tooth preparation, initial instrumentation of canals was done using a manual K file up to 15 No. along with 17% EDTA. Further instrumentation of canals was done using rotary endodontic files up to #25/6% using NeoEndo® Flex Rotary files. The canals were irrigated with 3 ml of 5% sodium hypochlorite (NaOCl) using a disposable, side-opened irrigating tip. At the end of instrumentation, the final irrigation of each sample was done using 5 ml of distilled water, and samples were dried using 6% paper points. All the prepared 56 root samples (n=56) were randomly divided into four groups depending on the intracanal medicament used, as shown in Figure 1.

Each of these intracanal medicaments containing inorganic metal oxide nanopowders (NPs) was mixed with vehicle polypropylene glycol 400 (PPG400) in the ratio of 1:3 p:w using a glass slab for 40 seconds to form a homogenous paste. A canal orifice was sealed using CaviTemp® temporary filling material to form a thickness of 1 mm after the placement of study materials (intracanal medicament) within the canals. Later, these teeth samples were stored in an incubator at 37ºC and 100% relative humidity for seven days. Following incubation, CaviTemp® temporary filling material was removed from the orifice, and canal instrumentation was done using the master apical file. The final rinse of prepared teeth samples was done using 5 ml of distilled water. The canals were dried using 25 No. 6% paper points and obturated using 25 No. 6% gutta-percha along with AH PlusTM (Dentsply International Inc., York, PA). The canal orifices were sealed using Fusion I-Seal®: light-curing glass ionomer cement at 1 mm thickness followed by light curing for 30 seconds. The prepared teeth samples were coated with double layers of nail varnish except in the apical 2/3rd of the root.

**Figure 2:**
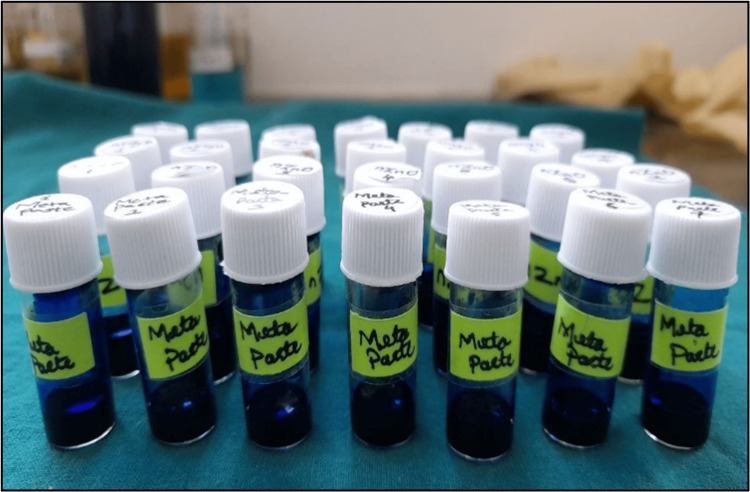
Pictorial representation showing prepared teeth samples immersed in 2% methylene blue dye solution

Further, these samples (n=56) with different experimental intracanal medicaments were randomly divided for qualitative (n=28) and quantitative (n=28) analysis to check the sealing integrity of newer nano inorganic metal oxide NPs in comparison with metapaste (control). After this, samples were stored in separate borosilicate glass and were incubated at 37°C at 100% relative humidity for seven days until the sealer was set completely. After verifying the complete setting of the AH PlusTM, each tooth specimen was immersed separately in an airtight container filled with 2% methylene blue dye for two days, as seen in Figure 2.

After two days, the specimens were rinsed under running tap water until no visible dye was appreciated. The prepared teeth samples were dried using tissue paper, and two coats of nail varnish were removed using cotton dipped in a 99% extra pure acetone solution.

Determination of sealing integrity using qualitative assay by measuring the total depth of penetration of dye

**Figure 3:**
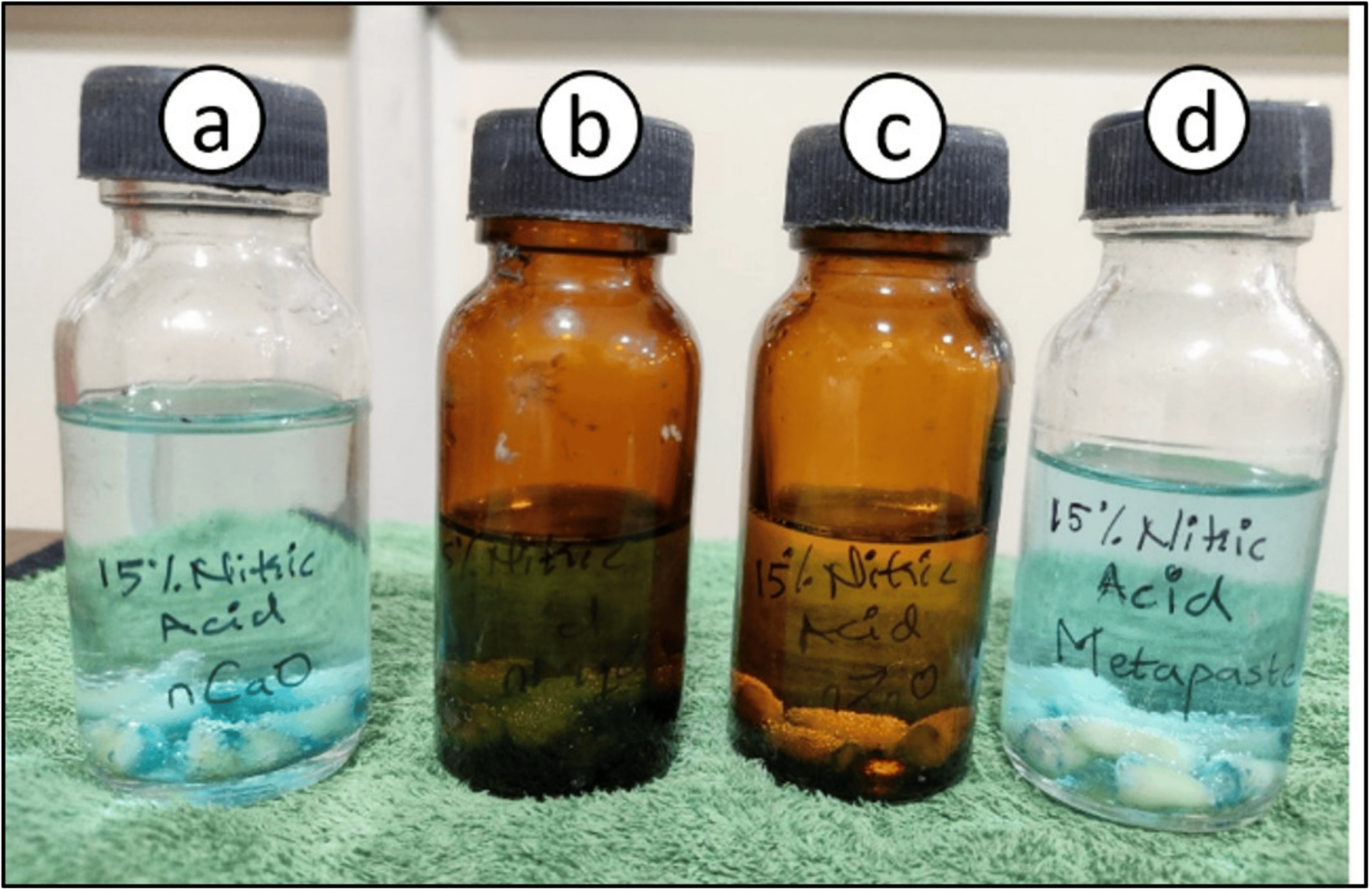
Pictorial representation showing an airtight container with teeth samples immersed in a 15% nitric acid solution containing test materials (a) nCaO, (b) nZnO, (c) nMgO, and (d) metapaste nCaO: nano calcium oxide; nZnO: nano zinc oxide; nMgO: nano magnesium oxide

A total of 28 prepared teeth samples (n=7 in each study group) were demineralized using 15% nitric acid for 24 hours, as seen in Figure 3.

**Figure 4:**
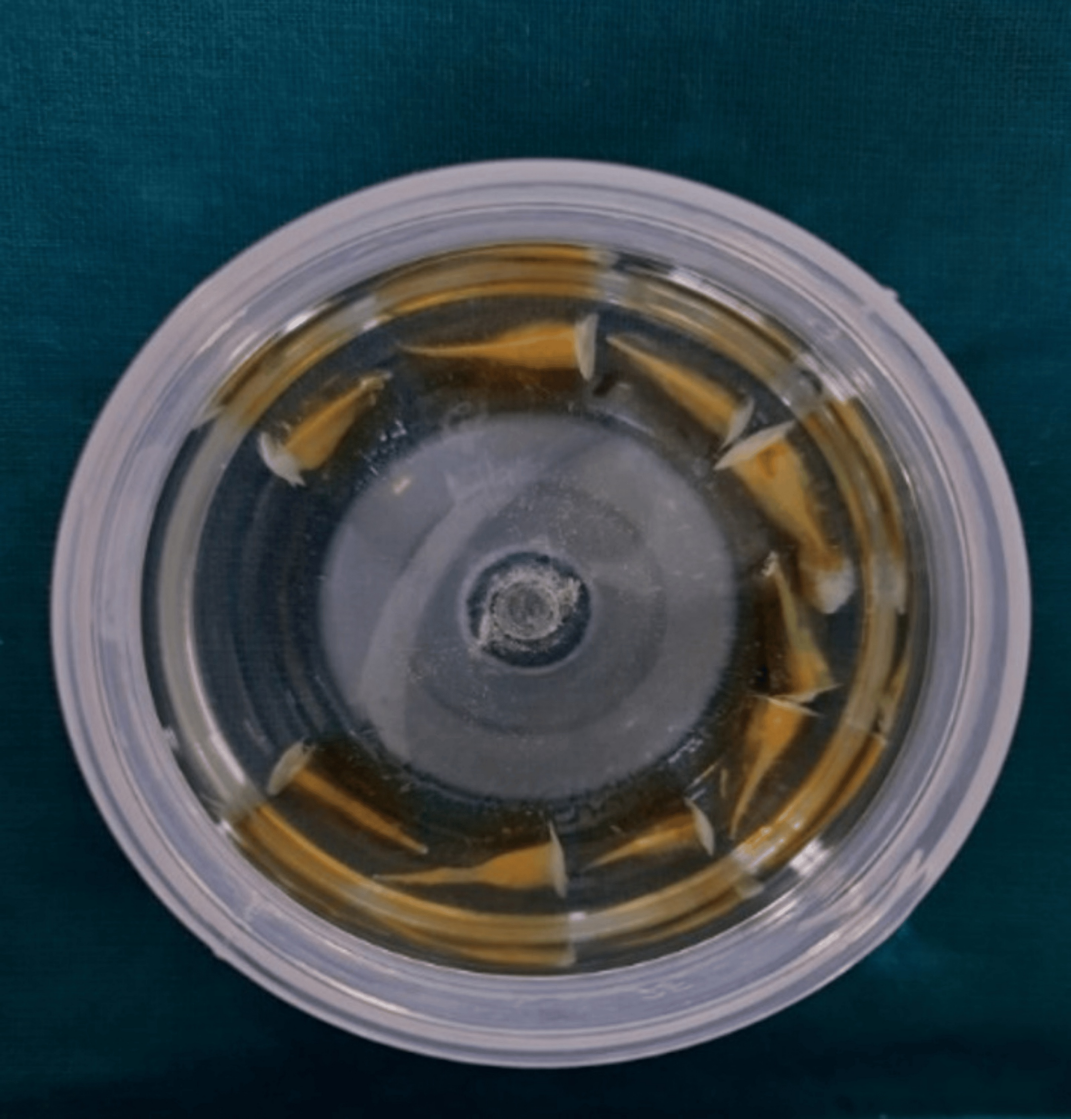
Pictorial representation showing diphanization of prepared teeth samples using methyl salicylate

Following demineralization, the prepared samples were washed under running tap water for four hours. The samples were dried with tissue paper, followed by the cycle of dehydration performed in ascending order of alcohols. The teeth samples were immersed completely in 80% ethyl alcohol for an overnight period, followed by 90% ethyl alcohol for one hour and absolute alcohol for three hours. A diphanization of teeth samples was done using methyl salicylate for 2-8 hours until teeth were cleared completely and appeared transparent, as seen in Figure 4.

**Figure 5:**
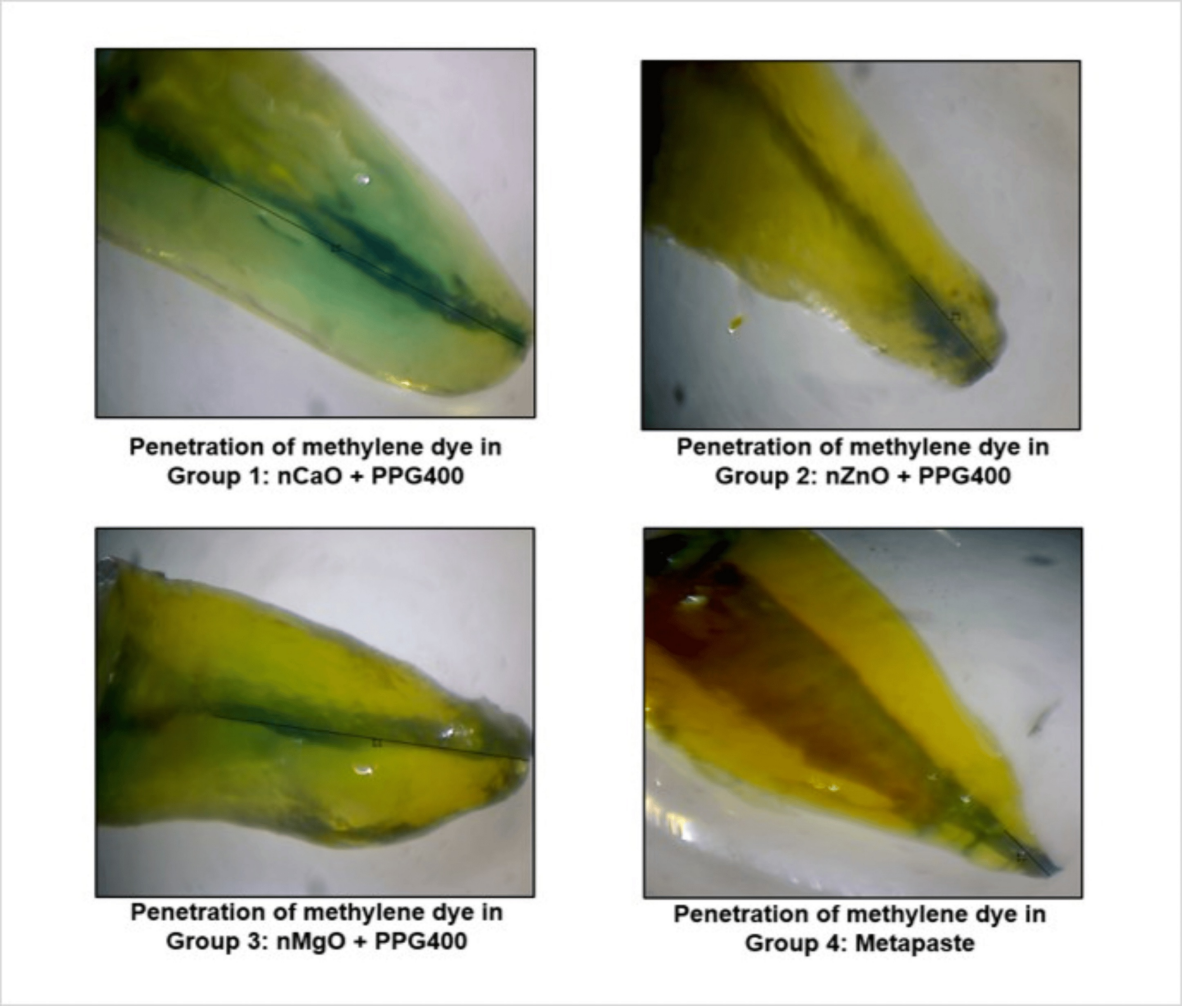
Pictorial representation showing stereomicroscope images of teeth samples with different groups showing the depth of dye penetration measured using Caliper Pro Image Software nCaO: nano calcium oxide; nZnO: nano zinc oxide; nMgO: nano magnesium oxide

The microleakage within the root canal was studied using a (Model SMZ171 Zoom, RRM Biotechnology, India) stereomicroscope at 45x magnification. The depth of dye penetration was measured by evaluating the linear extension of dye from the apical end of the root to the most coronal area that the dye had reached using Caliper Pro Image Software (Radical Scientific Equipments Pvt. Ltd., India) in millimeters (mm), as seen in Figure 5.

All the readings were documented using the following grading/scoring scale: Grade 0: Penetration of dye nil, grade 1: penetration of dye starts from the apical foramen and extends coronally between 0.1 and 1 mm; grade 2: penetration of dye starts from apical foramen and extends coronally between >1.1 and 2 mm; grade 3: penetration of dye starts from apical foramen and extends coronally between >2.1 and 3 mm; grade 4: penetration of dye starts from apical foramen and extends coronally between >3.1 and 4 mm; grade 5: penetration of dye starts from apical foramen and extends coronally between >4.1 and 5 mm; grade 6: penetration of dye starts from apical foramen and extends coronally between >5.1 and 6 mm; and grade 7: penetration of dye starts from apical foramen and extends coronally between >6.1 and 7 mm.

Determination of sealing integrity using quantitative assay by measuring the total amount of dye absorbed

**Figure 6:**
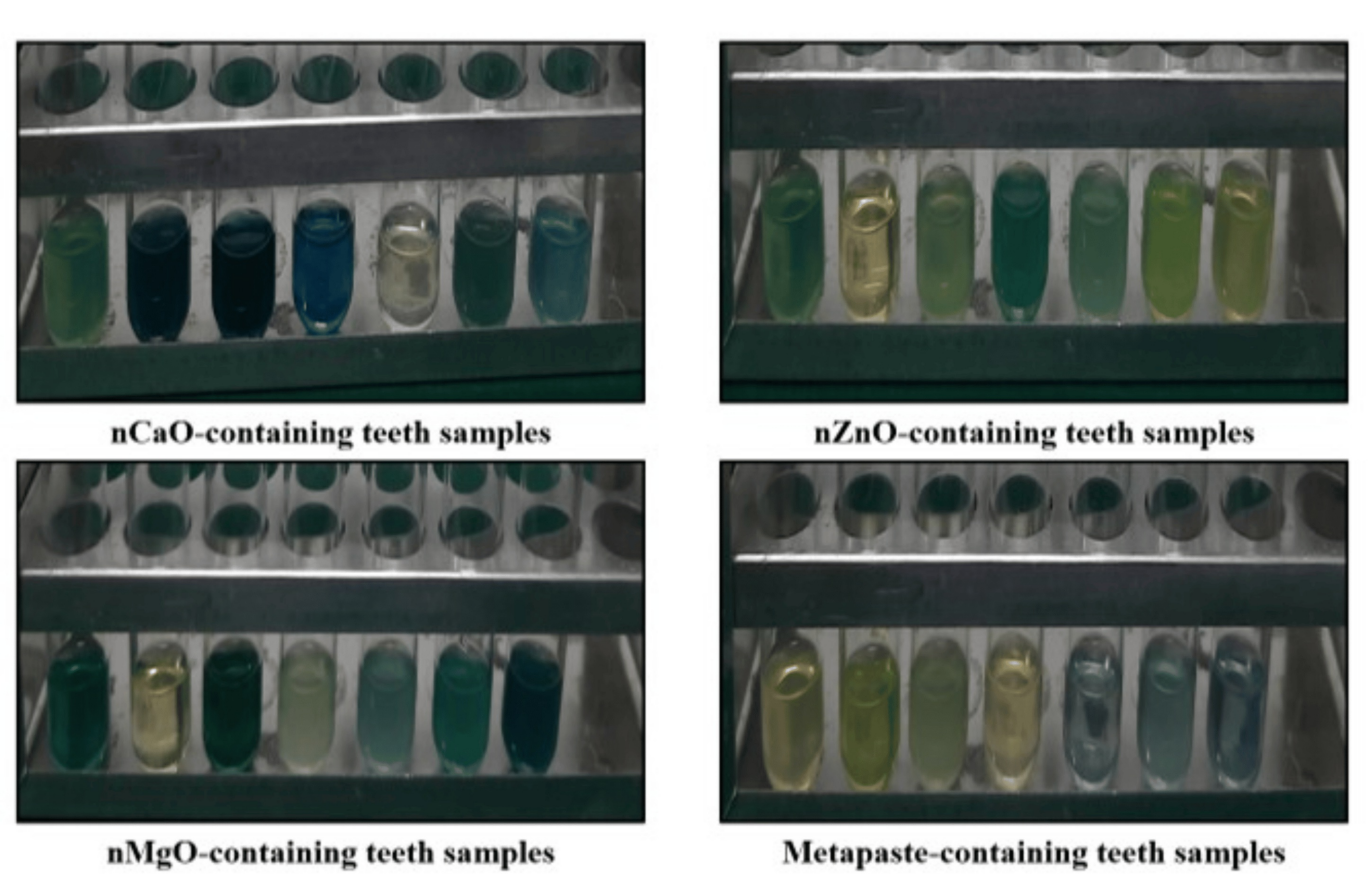
Pictorial representation shows teeth samples with study materials immersed in vials containing 69% HNO3 solution nCaO: nano calcium oxide; nZnO: nano zinc oxide; nMgO: nano magnesium oxide; HNO_3_: nitric acid

All 28 prepared teeth samples (n=7 in each study group) were separately stored in a hermetically sealed vial. To this, 1 ml of 69% concentrated nitric acid (HNO_3_) was added into these vials, and teeth, including gutta-percha and AH plus sealer, were allowed to completely dissolve for four days, as seen in Figure 6.

**Figure 7:**
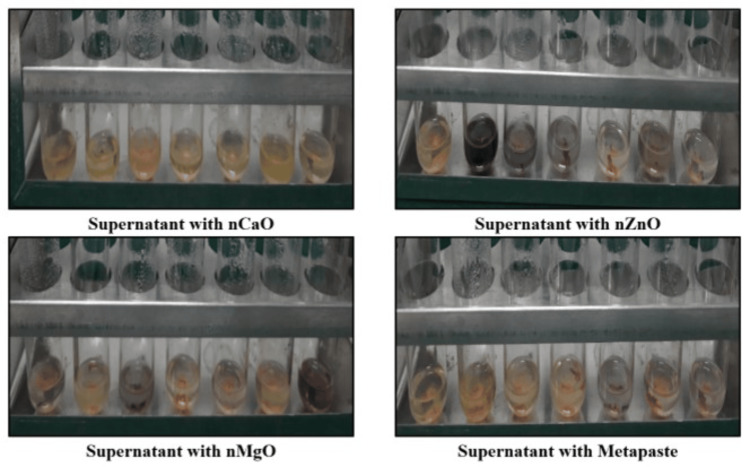
Pictorial representation showing supernatant on the centrifugation of vials containing teeth samples with test materials immersed in 69% HNO3 solution nCaO: nano calcium oxide; nZnO: nano zinc oxide; nMgO: nano magnesium oxide; HNO_3_: nitric acid

On the fifth day vials were centrifuged at 10000 rpm for 10 minutes. The supernatant formed on centrifugation as seen in Figure 7 was taken out using a micropipette and transferred into a vial for determination of the absorbance of 2% methylene blue dye using a spectrophotometer at 550 nm (Model LAB INDIA™ UV-3200 Double-beam, TechnoValue Solutions Pvt. Ltd., Navi Mumbai, India).

All the data was recorded, tabulated, and entered in Microsoft Excel (v. 2021, Microsoft Corporation, Redmond, United States). Statistical analysis was performed using IBM SPSS Statistics for Windows, Version 20 (Released 2011; IBM Corp., Armonk, New York, United States). Descriptive statistics were performed for experimental parameters assessing amongst experimental groups. The one-way analysis of variance (ANOVA) was applied to determine the qualitative assay by measuring the total depth of penetration of the dye. All statistical tests were performed at 95% confidence intervals, keeping a p-value of less than 0.05 as statistically significant.

## Results

**Figure 8:**
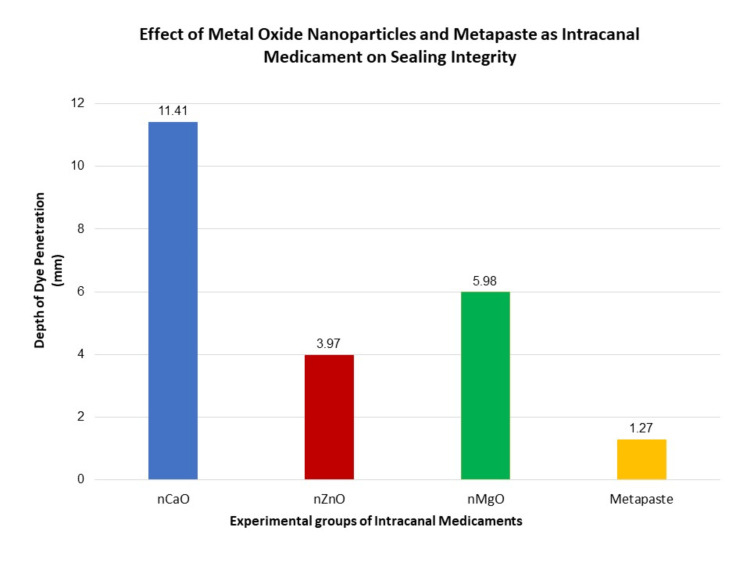
Graphical representation of the depth of dye penetration using a stereomicroscope

Table 1 and Figure 8 present the results of sealing integrity by evaluating the depth of dye penetration in teeth samples after immersion in a 2% methylene blue dye solution. It was observed that group 1 (nCaO) showed the maximum linear measurements of dye penetration with an average of 11.41 mm and SD of 1.35 within the interface of gutta-percha coated with sealer and teeth surface. Group 2 (nZnO) showed a mean average of 3.97 mm and SD of 10.56, less than group 3 (nMgO) with 5.98 mm and SD of 0.81. The least dye penetration was observed with group 4 (metapaste), with a mean average of 1.27 mm and SD of 0.63, with each group displaying statistically significant changes (p=0.0001).

**Table 1:** Descriptive statistics and one-way ANOVA test SD: standard deviation; ANOVA: analysis of variance; nCaO: nano calcium oxide; nZnO: nano zinc oxide; nMgO: nano magnesium oxide *significance inferred at p≤0.05 based on one way-ANOVA

Sealing Integrity	Depth of dye penetration (mm)		F-value	p-value
	Mean	SD		
Group 1 - nCaO	11.41	1.35	164.62	<0.0001*
Group 2 - nZnO	3.97	0.56		
Group 3 - nMgO	5.98	0.81		
Group 4 - Metapaste	1.27	0.63		

It was observed that in Table 2, according to the Student t-test, multiple comparisons of groups based on linear measurement of the depth of dye penetrated within the root canal system, we found statistically significant differences when group 4 (metapaste) was compared with group 1 (nCaO) (p<0.0001), group 3 (nZnO) (p<0.0001), and group 4 (nMgO) (p<0.0001). The comparison between group 1 (nCaO) and group 3 (nZnO) (p=0.0002) showed a significant difference. When group 1 (nCaO) was compared with group 3 (nMgO) and on comparison between group 2 (nZnO) with group 3 (nMgO) a statistically significant difference was observed with dye penetration (p<0.0001).

**Table 2:** Descriptive statistics and t-test SD: standard deviation; t-test: Student's t-test; nCaO: nano calcium oxide; nZnO: nano zinc oxide; nMgO: nano magnesium oxide *significance inferred at p<0.0001 based on t- value

Sealing integrity	Depth of dye penetration (mm)		t-value	p-value
	Mean	SD		
Metapaste	11.41	1.35	9.11	<0.0001*
nCaO	5.99	0.81		
Metapaste	11.41	1.35	13.45	<0.0001*
nZnO	3.98	0.56		
Metapaste	11.41	1.35	18.26	<0.0001*
nMgO	1.27	0.58		
nCaO	5.99	0.81	5.4	0.0002*
nZnO	3.98	0.56		
nCaO	5.99	0.81	12.54	<0.0001*
nMgO	1.27	0.58		
nZnO	3.98	0.56	8.89	<0.0001*
nMgO	1.27	0.58		

**Figure 9:**
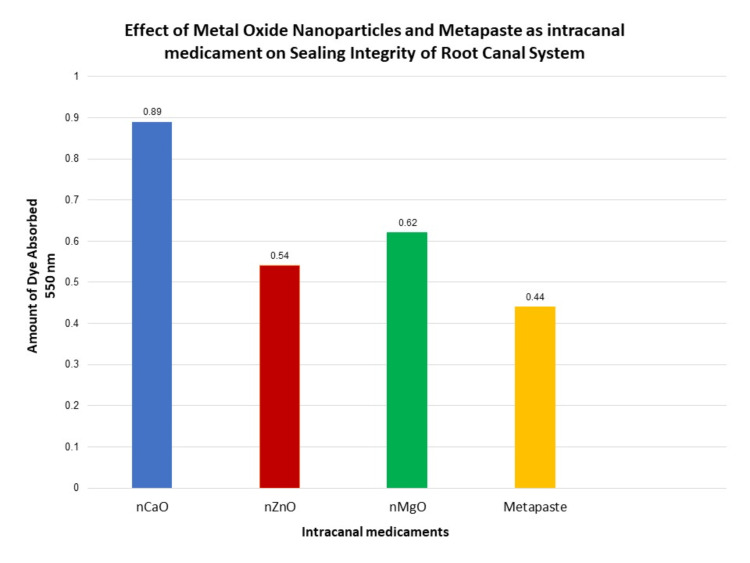
Graphical representation of the amount of dye absorbed using a spectrophotometer

Further Table 3 and Figure 9 present a quantitative assay used to determine sealing integrity by evaluating the total amount of dye absorbed in teeth samples. It was observed that group 1 (nCaO) showed maximum absorbance of 2% methylene blue dye with an average of 0.89 and SD of 0.63 at 550, while in group 2 (nZnO), a mean average of 0.54 and SD of 0.26 were present, which was less in comparison to group 3 (nMgO), with a mean average of 0.62 and SD of 0.29. The least absorbance of dye was observed with group 4 (metapaste) with a mean average of 0.44 and SD of 0.23, which was nonsignificant (p=0.17).

**Table 3:** Descriptive statistics and one-way ANOVA test SD: standard deviation; ANOVA: analysis of variance; nCaO: nano calcium oxide; nZnO: nano zinc oxide; nMgO: nano magnesium oxide Non-significant difference (p>0.0001)

Sealing integrity	Amount of dye absorbed (550 nm)		F-value	p-value
	Mean	SD		
Group 1 - nCaO	0.89	0.63	1.8	0.17
Group 2 - nZnO	0.54	0.26		
Group 3 - nMgO	0.62	0.29		
Group 4 - Metapaste	0.44	0.23		

It was observed that in Table 4, according to the Student t-test, multiple comparisons of groups based on the absorbance of 2% methylene blue dye solution within the root canal system, we found non-significant differences when group 4 (metapaste) (control) was compared with all the study groups. (p>0.0001). Further, when group 1 (nCaO) was compared with group 2 (nZnO) and group 3 (nMgO), non-significant results were observed. Also, when group 2 (nZnO) and group 3 (nMgO) were compared, the p-value was 0.23, which was statistically non-significant (p>0.0001).

**Table 4:** Descriptive statistics and t-test SD: standard deviation; t-test: Student's t-test; nCaO: nano calcium oxide; nZnO: nano zinc oxide; nMgO: nano magnesium oxide Non-significant difference (p>0.0001)

Sealing integrity	Amount of dye absorbed (nm)		t-value	p-value
	Mean	SD		
Metapaste	0.9	0.63	1.39	0.19
nCaO	0.54	0.26		
Metapaste	0.9	0.63	1.04	0.32
nZnO	0.62	0.3		
Metapaste	0.9	0.63	1.44	0.18
nMgO	0.44	0.23		
nCaO	0.54	0.26	0.53	0.6
nZnO	0.62	0.3		
nCaO	0.54	0.26	0.76	0.46
nMgO	0.44	0.23		
nZnO	0.62	0.3	1.26	0.23
nMgO	0.44	0.23		

## Discussion

*E. faecalis* is the most resistant microorganism causing endodontic flare-ups. The complete elimination of microorganisms from the root canal system is essential for endodontic success. Owing to the tortuous and ribbon-shaped root canal space, if left empty, would allow microbial recolonization. Hence it necessitates a need for complete sterilization of the pulp chamber using a cocktail of procedures that include biomechanical preparation, irrigation, and intracanal medicaments. The present study focuses on evaluating the sealing integrity of root canal-treated teeth that were exposed to NP-based intracanal medicaments.

The study performed by Ricucci and Langeland (1997) and Kim and Kim (2002) reported difficulty in the complete removal of Ca(OH)_2_-based intracanal from the root canal before obturation. This might be due to the presence of remnants of intracanal medicaments that may interfere with the integrity of sealing of permanent obturating material with a sealer to canal walls, along with this intracanal medicament may occlude dentinal tubules preventing sealer penetration. These medicaments, due to their solubility, degrade over time, causing gaps that could form niches for the colonization of bacteria. This compromises the success of the endodontic treatment [2,3]. Lambrianidis et al. (1999) reported that none of the existing techniques are efficient in removing the entire intracanal medicament from canals and leave up to 45% of the root canal surface covered with remnants of medicament which may interact with the root canal sealer and interfere with its sealing ability [4].

In the study conducted by Barge et al. (2023), the order of proliferative activity of experimental nano intracanal medicaments: meta paste > nCaO > nMgO > nZnO was observed on L929 mouse fibroblast cells using MTT assay [5]. On comparison of antimicrobial activity, nCaO showed maximum zone of inhibition and minimum inhibitory concentration against *E. faecalis* compared to nMgO, nZnO, and metapaste. As these novel nano medicaments presented with maximum antimicrobial activity against the most resistant microorganism, *E. faecalis, *and showed a minimum cytotoxic effect on fibroblast cells, the present study further evaluates the sealing integrity of an endodontically treated tooth using these novel NPs based intracanal medicaments. For standardization purposes, the NPs used as an intracanal medicament in the current study ranged between 20 and 40 nm.

Nalawade et al. (2015) compared the antimicrobial activity of five vehicles that included propylene glycol, glycerin, PEG 400, PEG 1000, and a combination of propylene glycol with PEG 400 against four standard strains of organisms that included *Streptococcus mutans* American Type Culture Collection (ATCC) 25175, *Streptococcus mutans* ATCC 12598, *E. faecalis* ATCC 35550, and *Escherichia coli *ATCC 25922, using broth dilution assay. The study concluded that all experimental vehicles showed bactericidal activity at 100% concentration. Propylene glycol had a bactericidal effect at a concentration of 50% on *S. mutans*, 25% on *E. faecalis*, and 50% on *E. coli*. Vehicle PEG 1000 was effective against *S. mutans* and *E. coli* at 25% [6]. Further, in the experimental study by Ashraf et al. (2019), various ratios of liquid (coconut oil) and powder (CaO) like 1:1, 1:2, 1:3, 1:4, 1:5, 1:6, 2:1, 3:1, 4:1, 5:1, and 6:1 were used in which best outcomes were observed with powder/liquid ratio of 1:3 without additives when compared with metapaste [7]. For standardization purposes in this present study, the vehicle used was propylene glycol owing to its antimicrobial activity against the most resistant* E. faecalis*. The control metapaste used in this experimental study has polypropylene 400 (PPG400) as a vehicle. Hence, it was considered a vehicle during the manipulation of the NPs to form the experimental intracanal medicaments. During the pilot study, a powder/liquid ratio of 1:3 was observed on the viscometer (Brookfield, USA), when the viscosity of novel NP-based intracanal medicament was compared with that of metapaste.

The dehydration (diphanization) technique allows a 3D view of the internal anatomy of root canals within the root with no loss of the tooth substance, facilitating visualization of the leakage area. This simple procedure enhances the visualization of lateral and accessory canals revealing the relationship between the sealing material and the tooth interphase. The drawback of this technique is seen when the demineralization of the tooth, if not done completely, causes compromised visualization of dye penetration within the tooth specimen. This technique is more accurate than transverse sectioning of the tooth sample for detecting apical leakage as it reveals leakage in fractions of a millimeter, while transverse sectioning only determines the presence or absence of leakage, so in the current study, the dehydration (diphanization) technique was preferred. Thus, perfect apical sealing is desirable for preventing bacterial infiltration into the root canals. A similar technique was used by Sokhi et al. (2017) to check the effect of Ca(OH)_2_ intracanal medicament on the sealing ability of the sealer and gutta-percha [8].

Various methods like dye selection, dye extraction/dissolution method, fluid filtration/transportation method, glucose penetration model using fluid filtration, and radioisotope penetration method are used to determine the sealing integrity [9]. Amongst these, dye selection and dye extraction/dissolution methods are most commonly used and are less technique-sensitive and cost-effective. These assays can be performed using various agents like methylene blue dye, butyric acid, rhodamine B, and India ink. In the present study, the methylene blue dye penetration methodology was used as it is easy to perform and has a relatively low molecular weight. Methylene blue is a more sensitive indicator of leakage than other dyes. It also facilitates easy penetration into accessory canals and dentinal tubules due to its passive capillarity phenomenon as the tooth apex is submerged in the dye [10].

In the current study, freshly extracted human teeth were used to improve reliability by simulating the clinical situation. A tooth with straight roots, mature apices, and single patent root canals was selected to minimize anatomical variation and allow standardization. A decoronization was performed and the radicular part of the tooth was sectioned in a transverse plane 15 mm from the apex toward the coronal part to facilitate instrumentation and allow standardization. In the present study, intracanal medicament was given for seven days which was disharmonious to Nerwich et al. (1993), where the medicament was placed for 14 days which is the interval between appointments as hydroxyl ions derived from a Ca(OH)_2_ intracanal dressing diffuse in an hour into the radicular dentin while it requires 2-3 weeks to reach peak levels. The use of calcium oxide (CaO) as intracanal medicaments was done as an alternative to Ca(OH)_2_ in the study by Ashraf et al. (2019) where CaO-based intracanal medicaments showed a lower apical leakage when compared to Metapex. This may be due to the very little solubility of CaO and its ability to dissolve the organic materials within dentinal tubules that aid the sealer to penetrate deep inside dentinal tubules and few remnants of CaO remain within the dentinal tubules for a time, which will cause precipitation of Ca within dentinal tubules which further enhances intimate apical sealing preventing microleakage [7,11]. Whereas in the present study, the amount of methylene blue penetrated within the root canals and the actual amount of dye absorbed was highest with nCaO which was highest when compared to metapaste.

Porkaew et al. (1990) studied the effects of Ca(OH)_2_ remnants along the canal walls on the sealing ability of the permanent filling material. Seventy-six extracted human teeth were decoronated and samples were randomly divided into group 1 (Ca(OH)_2_ USP), group 2 (Calasept), group 3 (Vitapex), and group 4 (control) (n=18 each). The samples were examined under the scanning electron microscope after seven days followed by gutta-percha obturation. The prepared root samples were placed in a 2% methylene blue solution for two weeks after which they were subjected to linear and volumetric dye penetration evaluation. Results showed a highly significant correlation between linear and volumetric leakage. It was concluded that there is a significant decrease in leakage of medicated teeth using Ca(OH)_2_ [12]. In the experiment performed by Tandan (2014), tooth samples were immersed in the dye for three days and only the amount of dye absorbed was measured whereas the depth of penetration was not taken into consideration [13]. On the contrary, in the present study, the tooth was immersed in a methylene blue dye solution for two days owing to the capillary action, and both qualitative and quantitative assays were performed to evaluate the sealing integrity. In the current study, minimum dye penetration was observed with metapaste followed by nZnO and nMgO, and maximum dye was absorbed with nCaO. This may be possible as metapaste being an oil-based medicament might have blocked the entry of methylene blue dye within the root canals when compared to NPs. Another possible reason could be the short duration of the placement of the medicament.

Louwakul et al. (2017) placed a total of 50 µl of CaO NPs and Ca(OH)_2 _NPs on the tooth surface where antimicrobial efficacy was evaluated using fluorescent staining and confocal laser scanning microscopy (CLSM) to confirm the effect of the intracanal medicaments on the *E. faecalis* in the dentinal tubules. The author concluded that Ca(OH)_2_ NPs were more efficient than CaO NPs in the elimination of *E. faecalis* in the dentinal tubules (p<0.05). The control groups that included CaO and Ca(OH)_2_ didn’t show a bactericidal effect. Hence, this study concluded that NPs can be effectively used in the elimination of *E. faecalis* in dentinal tubules [14].

Limitations

The voids formed due to trapped air within the obturation material concerning the dye penetration that might have interfered with fluid movement. Kontakiotis et al. (2001) concluded that methylene blue penetrated along air-filled gaps by capillary action, while it penetrated water-filled gaps by diffusion [15]. An important consideration concerning the depth of dye penetrated and the total amount of dye absorbed are the molecular size, pH, and chemical reactivity, which are not considered. The dye extraction technique presents a drawback in which the apical 2 mm of the tooth sample was exposed to methylene blue dye and was not double coated with varnish, permitting dye to penetrate the apical region of dentin that was dyed blue even after washing the specimen under running tap water. Further, this experimental in vitro study did not consider the role of the vehicle (PPG400) in the dye penetration within the root canals. As a result, additional research is needed to estimate these resources to authenticate the in vitro-recognized outcomes.

## Conclusions

Within the limitations of this study, it was observed that all teeth samples after the use of intracanal medicament exhibit some amount of dye penetration within the root canal system. Amongst all the groups, metapaste (control) shows minimal linear dye penetration and exhibits a minimum amount of dye absorbed when compared to a novel NP-based formulation of intracanal medicaments. Within the experimental group, nZnO+PPG400 and nMgO+PPG400 showed better results than nCaO+ PPG400; hence, it can be a newer alternative to metapaste.

This is the first study to measure the sealing integrity of these novel metal oxide NP-based intracanal medicaments in combination with PPG400 as a vehicle for methylene blue dye assays and lacks evidence in the literature. Hence, further studies are required to validate the role of PPG400 as a vehicle and 2% methylene blue dye on the sealing integrity of the root canal system.
